# Temperature-robust neural activity using feedback control of ion channel expression

**DOI:** 10.1186/1471-2202-15-S1-P103

**Published:** 2014-07-21

**Authors:** Timothy O’Leary, Eve Marder

**Affiliations:** 1 Volen Center for Complex Systems, Brandeis University, Waltham, MA 02454, USA

## 

Neural activity depends on the kinetic properties of ion channels expressed in neurons. Small changes in these properties can dramatically affect synaptic integration, membrane excitability and circuit function. Like all biochemical processes, the kinetics of ion channels have an exponential temperature dependence and the exponent (the ‘Q_10_’) differs several-fold between ion channel types within species [1-3]. In warm-blooded animals such as mammals, deviations in temperature of only a few degrees Celsius can thus disrupt neural activity and lead to loss of consciousness or death. However, cold blooded animals, including all invertebrates, manage to survive and function despite temperature fluctuations of tens of degrees Celsius [1]. How is this robustness achieved? One possibility is that the self-regulating, activity-dependent mechanisms that maintain neuronal properties in cold-blooded animals operate in a way that specifically gives rise to temperature robustness. In this work we develop a model of activity-dependent ion channel regulation that can produce stable neuronal activity in spite of underlying differences in the temperature dependence and density of conductances in model neurons. We use this model to explore the constraints that temperature-robustness imposes on self-regulating models and the resulting consequences for neuronal properties and circuit function.
